# Circulating Immune Complexes and Complement Activation in Sensitized Kidney Transplant Recipients

**DOI:** 10.3390/ijms252010904

**Published:** 2024-10-10

**Authors:** Maria Stella Trivyza, Charikleia Stergiopoulou, Sotiris Tsakas, Theodoros Ntrinias, Marios Papasotiriou, Nikolaos Karydis, Evangelos Papachristou, Dimitrios S. Goumenos

**Affiliations:** 1 Department of Nephrology and Kidney Transplantation, University Hospital of Patras, 26504 Patras, Greece; mariastella421@gmail.com (M.S.T.); th.ntrinias@gmail.com (T.N.); epapachr@upatras.gr (E.P.); 2 Laboratory of Biology, Department of Biology, University of Patras, 26504 Patras, Greece; up1073878@ac.upatras.gr (C.S.); stsakas@upatras.gr (S.T.); 3 Department of Surgery, University Hospital of Patras, 26504 Patras, Greece; nkarydis@upatras.gr

**Keywords:** immune complexes, CH50, kidney transplantation, donor-specific antibodies

## Abstract

Chronic antibody-mediated rejection in kidney transplantation is a common cause of graft loss in the late post-transplant period. In this process, the role of the classical complement activation pathway is crucial due to the formation of immune complexes between donor-specific antibodies (DSAs) and donor antigens and the attachment of the C1q complement fragment. This study aimed to determine the levels of circulating C1q immunocomplexes (CIC-C1q) and complement activation (CH50), in sensitized kidney transplant recipients (KTRs). In this cross-sectional study we used serum samples from KTRs with de novo or preformed DSAs (*n* = 14), KTRs without DSAs (*n* = 28), and 22 subjects with no history of chronic kidney disease (controls). C1q immunocomplexes and CH50 concentration in serum were measured with the enzyme immunoassay (EIA) kit MicroVue CIC-C1q (Quidel, Athens, OH, USA) and EIA kit MicroVue CH50 (Quidel, OH, USA), respectively. Higher concentrations of CIC-C1q was observed in KTRs with DSAs in comparison with controls and with KTRs with no DSAs (6.8 ± 2.7 and 4.8 ± 1.9 vs. 5.0 ± 1.2 μg Eq/mL, respectively, *p* < 0.01). We found no difference in CIC-C1q between KTRs with no DSAs and controls. CIC-C1q levels were positively correlated with DSA titer. CH50 levels were decreased in KTRs with DSAs in comparison with controls and KTRs with no DSAs (39 ± 15 vs. 68 ± 40 and 71 ± 34 U Eq/mL, respectively, *p* < 0.01). There was no difference in CH50 between DSA-negative KTRs and controls. Kidney transplant recipients with DSAs had increased serum levels of C1q immunocomplexes and increased classical pathway complement activation.

## 1. Introduction

Hemodialysis, peritoneal dialysis, and kidney transplantation are the main treatment options for end-stage kidney disease. Among these, kidney transplantation is highly associated with improvement of patients’ quality of life [1]. Kidney transplantation, though, is hindered by the development of donor-specific antibodies (DSAs) against human leukocyte antigens (HLAs). This is associated with increased incidence of antibody-mediated rejection (AMR), subsequent increased risk of graft loss, and increased patient mortality [2]. The development of de novo DSAs in previously non-sensitized KTRs occurs in approximately 30% of patients, according to previous studies. Specific risk factors for development of de novo DSAs include the increased number of HLA mismatches, low levels of immunosuppression or nonadherence to treatment, and factors that increase graft immunogenicity (graft inflammation or infection, cellular rejection, and ischemia injury). De novo DSAs mainly target donor HLA-II antigens and can appear any time after kidney transplantation, although this mostly occurs during the first year after surgery [3,4,5]. Specific epitopes that are located in the polymorphic regions of HLAs are targeted by DSAs. For HLA-I antigens (A, B, and C), which are expressed on all nucleated cells, epitopes reside only in the polymorphic a-chain but not in b2-microglobulin. For HLA-2 antigens (DR, DQ, and DP), which are expressed on antigen-presenting cells (dendritic cells, B cells, and macrophages), epitopes reside in either or both of the polymorphic a-chain and the polymorphic b-chain [6,7].

However, not all DSAs are of equal significance in terms of promoting acute or chronic AMR, with its devastating effects on allograft function, as it is shown by a significant percentage of patients with DSAs who ultimately show relatively stable graft function. Many reasons could explain this phenomenon, such as the use of different immunosuppressive regimens and the absence or presence of different comorbidities. Nevertheless the phenomenon has not been adequately explained. More importantly, the ability of DSAs to activate the complement may be a discriminating factor between clinically significant DSAs that induce AMR and those of low immunogenic significance. Timely detection and reliable monitoring of antibody-mediated rejection is a clinical challenge in everyday clinical practice. Ideally, the stratification of kidney transplant recipients (KTRs) based on a complement-activation biomarker could enhance clinical decision-making regarding AMR treatment [8]. 

Graft injury and AMR occur mainly through the activation of the classical complement pathway [9]. The classical complement-activation pathway involves the binding of the complement fraction C1q, specifically its globular domains, with specific IgG or IgM that, in turn, are bound to graft endothelial antigens [10]. This interaction between C1q and IgG or IgM leads to the sequential activation of C1r and C1s and initiates the activation of the complement cascade and the generation of the key effector molecule of the complement system, the terminal membrane attack complex, which causes cell lysis [11]. However, even if this key pathophysiological pathway is not activated there are certain complement-independent mechanisms that allow DSAs to cause kidney damage. This can happen either through antibody-dependent cellular cytotoxicity, or through direct activation of endothelial proliferation due to increased vascular endothelial growth factor production or upregulation of other signaling pathways of cellular recruitment [12,13].

Overall, not all DSAs have the same effect on allograft function [14,15] and a reason for this is that not all DSAs have the capacity to fixate C1q, activate the classical complement pathway, and promote allograft rejection [10,11]. In the present study, we investigated the correlation between post-transplant C1q-containing immune complexes (CIC-C1q) and total complement activity (CH50) in KTRs with or without DSAs.

## 2. Results

Forty-three KTRs were included in this study. In 15 of them, donor-specific antibodies (DSAs) were identified through the comparison of donor-recipient HLA mismatch to the antibody profile in each patient. No DSAs were detected in the remaining 28 KTRs. Therefore, there were nearly twice as many as KTRs without DSAs as with them. A third group of 22 healthy subjects with no previous history of kidney disease was used as a control group (normal controls). Patients’ and normal controls’ baseline clinical and biochemical characteristics are presented in Table 1. Comorbidities such as cardiovascular disease (coronary artery disease, cerebrovascular disease, and peripheral arterial disease) and diabetes mellitus in transplant recipients are presented in Supplemental Table S1.

During follow-up, the 15 DSA-positive patients experienced a significant deterioration in their kidney function (eGFR: 53.1 ± 23.8 vs. 41.1 ± 26.1 mL/min/1.73 m^2^, *p* < 0.001), with 3 of them reaching ESKD and starting maintenance hemodialysis. As far as the DSA-negative KTRs were concerned, 2 out of 28 patients reached ESKD and showed an overall significant decrease in kidney function during a follow up period of 19 months (61.4 ± 27.4 vs. 52.9 ± 27.1, mL/min/1.73 m^2^, *p* = 0.007).

### 2.1. Donor-Specific Antibodies and Kidney Function

Among the 15 DSA-positive patients, 6 had pre-formed DSAs and 9 developed de novo DSAs. Three cases of DSAs against HLA-I and twelve against HLA-II antigens were detected. By HLA loci, two DSAs were against HLA-A, one against HLA-B, two against HLA-Cw, four against HLA-DR, and six against HLA-DQ.

Kidney function (eGFR) of KTRs with and without DSAs did not differ significantly (53 ± 24 vs. 61 ± 27 mL/min/1.73 m^2^, *p* = 0.54) but were both lower in comparison with controls (106 ± 13 mL/min/1.73 m^2^, *p* < 0.001). 

### 2.2. Circulating Immune Complexes Correlations

**Figure 1:**
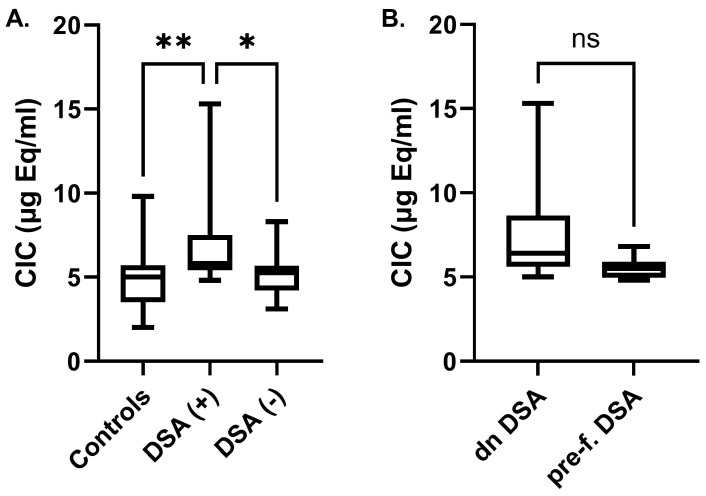
Circulating immune complexes (CICs) in healthy controls and kidney transplant recipients without (DSA−) or with (DSA+) donor-specific antibodies (**A**) and in KTRs with pre-formed or de novo DSAs (**B**). (*, *p* < 0.05, **, *p* < 0.01, ns: non-significant).

A significantly increased concentration of C1q-CIC was found in the serum of KTRs with DSAs, in comparison with controls as well as with the group of KTRs without DSAs (6.8 ± 2.7 vs. 4.8 ± 1.9 and 5.0 ± 1.2 μg Eq/mL, respectively, *p* < 0.01) (Figure 1A). There was no significant difference in C1q-CIC between DSA-negative KTRs and controls. Finally, the mean concentration of C1q-CIC in KTRs with de novo DSAs was higher compared with KTRs with pre-formed DSAs (7.6 ± 3.2 vs. 5.5 ± 0.7 μg Eq/mL, *p* = 0.09) (Figure 1B). Nevertheless, this did not reach statistical significance, probably due the limited number of samples.

No correlation was found between C1q-CIC and eGFR, lymphocyte count, neutrophil count, or neutrophil-to-lymphocyte ratio (NLR). Although NLR did not differ between DSA-negative and DSA-positive KTRs (2.6 ± 1.4 vs. 3.0 ± 1.7, *p* = 0.6), they were both higher in comparison with controls (1.6 ± 0.6, vs. DSA (−), *p* = 0.04 and vs. DSA (+), *p* = 0.008). 

**Figure 2:**
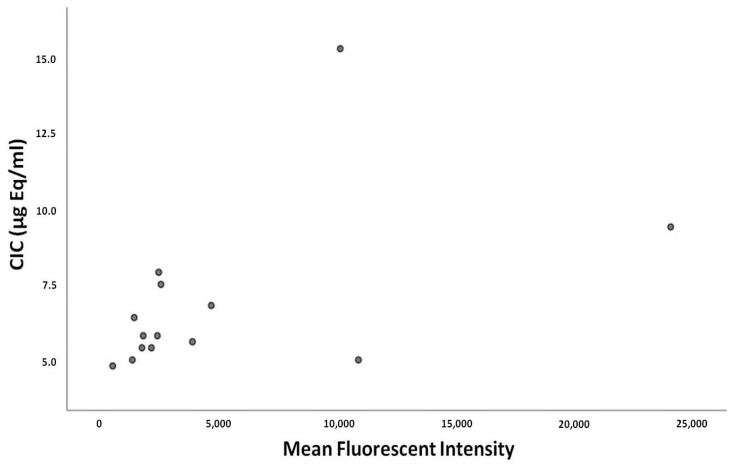
Correlation of circulating immune complexes (CICs) and donor-specific antibodies (mean fluorescent intensity).

We also examined whether the time from the kidney transplant procedure correlated with C1q-CIC levels, but we found no significant correlation (Spearman’s rho: 0.376, *p* = 0.186). Furthermore, no significant differences in tacrolimus trough levels were observed among DSA (+) patients and we found no correlation between C1q-CIC and tacrolimus levels (Spearman’s rho: 0.22, *p* = 0.43). Finally, and more importantly, C1q-CIC levels were positively correlated with DSA titer (r^2^: 0.239, *p* = 0.035) (Figure 2). 

### 2.3. Complement-Activation Correlations

**Figure 3:**
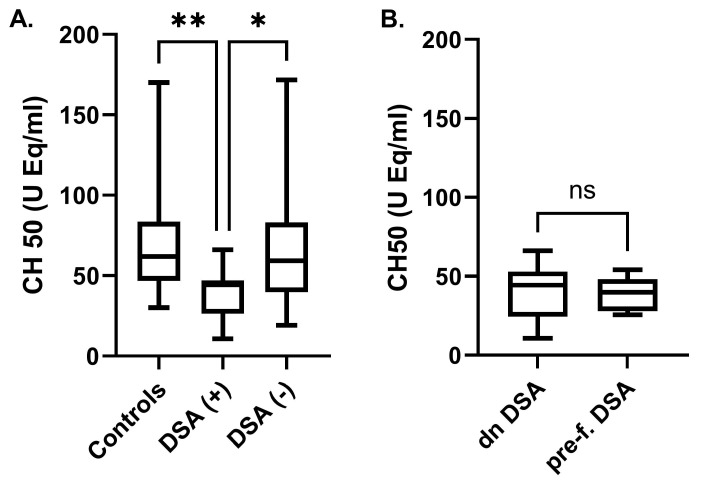
Complement activation (CH50) in healthy controls and kidney transplant recipients without (DSA−) or with (DSA+) donor-specific antibodies (**A**) and in KTRs with pre-formed or de novo DSAs (**B**). (*, *p* < 0.05, **, *p* < 0.01, ns: non-significant).

The increased activation of total complement in KTRs with DSAs compared with controls and KTRs with no DSAs was manifested through a significant reduction in CH50 levels in the former group (39.2 ± 15 vs. 70.6 ± 34.3 and 68 ± 39.9 U Eq/mL, respectively, *p* < 0.001), a finding which suggests higher levels of classical pathway complement activation (Figure 3A). CH50 levels in KTRs with DSAs, subcategorized into those with pre-formed and those with de novo DSAs, did not differ significantly (39.3 ± 17.8 vs. 39.1 ± 11.1 U Eq/mL, *p* = 0.98) (Figure 3B).

**Figure 4:**
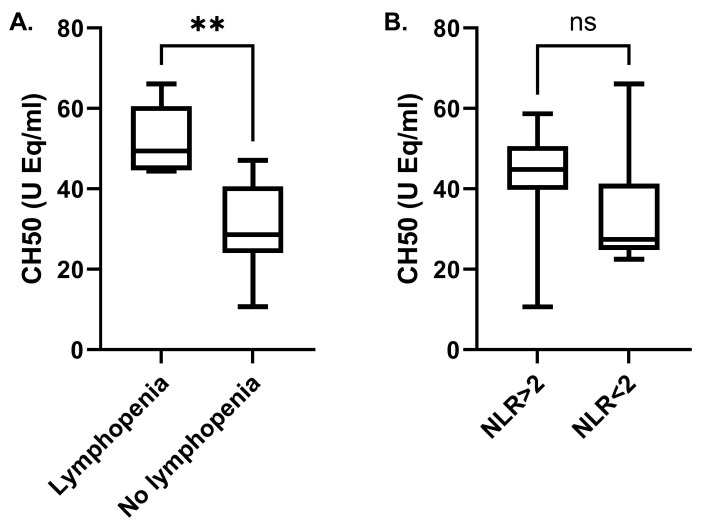
Complement activation (CH50) in KTRs in relation to lymphopenia (**A**) and the neutrophil to lymphocyte ratios (NLR) (**B**). (**, *p* < 0.01, ns: non-significant).

Apart from the reduced CH50 activity in DSA-positive KTRs, further analysis showed a significant relationship between subgroups according to lymphocyte count. The CH50 activity level was higher in patients with lymphopenia (lymphocyte count < 1500 cells/μL) compared with those without lymphopenia (lymphocyte count > 1500 cells/μL) (52.1 ± 9.1 vs. 30.6 ± 11.5 U Eq/mL, *p* < 0.01) (Figure 4A). No difference was observed when comparing neutrophil to lymphocyte ratios (NLR < 2:33.7 ± 16.3 vs. NLR > 2:42.9 ± 13.7 U Eq/mL, respectively, *p* = 0.15) (Figure 4B). 

## 3. Discussion

In this cross-sectional study we examined the difference in C1q immunocomplex concentrations and serum CH50 levels in three different groups of subjects: KTRs with either pre-formed or de novo DSAs, KTRs without DSAs, and healthy controls. We found that KTRs with DSAs show higher concentrations of C1q-CIC in comparison with controls as well as with DSA-negative KTRs. Moreover, our results indicated that C1q-CIC levels correlated positively with the DSA titer. This suggests that the classical complement pathway in patients with DSAs manifests higher levels of activation, as confirmed by the lower CH50 values found in DSA-positive KTRs. 

Acute rejection is common in patients after kidney transplant, especially during the early stages, but also during the late post-transplant period. Acute rejection episodes are either T-cell- or antibody-mediated. T-cell-mediated rejection is characterized by interstitial CD4+ and CD8+ T-cell infiltrates and subsequent inflammation that extends into tubules via injury to the tubular basal membrane producing the classical histological finding of tubulitis [16]. The production and presence of DSAs in the serum of KTRs is not a prerequisite for T-cell-mediated rejection. However, recent studies have exhibited that the presence of DSAs with C1q-binding capacity is associated with a higher incidence of T-cell-mediated rejection, when compared with patients with DSAs that are not able to bind C1q or patients without DSAs [17]. Thus, immunocomplexes formed from DSAs and C1q not only correlate with antibody-mediated rejection, as anticipated, but to T-cell-mediated rejection as well. Moreover, DSA production by the kidney transplant recipient has the potential of promoting activation of the classical complement pathway, as has already been shown in other studies [3,17,18] and confirmed in our cohort.

C1q-binding DSAs have been found to strongly correlate with DSA MFI levels [19]. However, it is not clear whether this feature is an independent risk factor for acute rejection or chronic allograft nephropathy and subsequent early graft loss. More specifically, pre-formed C1q-binding DSAs, in spite of their correlation with DSA MFI, were not associated with cumulative biopsy-proven acute rejection in one year, nor with cumulative graft survival [19]. Similar results have been observed with significantly higher MFI values in the C1q-positive group compared with the C1q-negative group [20]. It seems that, overall, DSA MFI values cannot be used as a surrogate indicator for determining the complement-activation capacity of HLA antibodies, as other studies have not proven this association [21,22]. In our study, we found that patients with higher DSA MFI titer had significantly higher C1q immunocomplexes in serum, indicating that patients with higher DSA MFI titers are more prone to classical complement activation. 

C1q-binding DSAs have been associated with a higher risk of kidney allograft loss and a lower 5-year graft survival rate [17]. Analysis of DSA characteristics is increasingly used to provide better immunological risk stratification for patients with pre-formed DSAs as well as for those with de novo DSAs. Regarding KTRs with pre-formed DSAs in particular, it has been shown that DSAs with increased ability to bind C1q convey an increased risk of AMR and lower graft survival [3]. Furthermore, in a study with more than 1000 included KTRs, patients with de novo C1q-binding DSAs had a significantly lower 5-year graft survival rate (54% vs. 93%, *p* < 0.001) [17]. In the same study, it was also found that development of de novo DSAs with C1q binding capacity was the worst clinical scenario in terms of graft survival, even compared with the presence of pre-formed C1q-binding DSAs [17]. It is mainly this particular feature of DSAs, their ability to induce classical complement activation, that carries a greater potential to serve as a predictor of rejection in comparison with DSA MFI levels alone [3]. 

Activation of the classical complement pathway, as noted in several clinical situations (e.g., systemic lupus erythematosus or immune-complex-associated glomerulonephritis), is commonly associated with low serum levels of CH50 [23]. Concerning KTRs, in a large cross-sectional study with more than 100 DSA-positive KTRs, serum CH50 levels were not different between DSA-positive and DSA-negative patients. More importantly, complement patterns in serum did not overall offer any diagnostic value in relation to AMR [24]. On the contrary, in this cohort we observed that DSA-positive KTRs had a significantly lower serum level of CH50, which is indicative of classical complement pathway activation. 

We also examined whether NLR could be utilized as a marker, or as a readily available indicator of complement activation. Concerning lymphopenia and serum complement activation, as expressed by low CH50 values, a decreased lymphocyte count can be an indicator of poor prognosis in several conditions, including patients after liver transplantation [25]. Furthermore, the prognostic value of NLR has been reported in conditions such as coronary artery disease, solid tumors, and rheumatoid arthritis [26,27,28]. Moreover, NLR has been reported to show an association with the prognosis of kidney diseases, including acute kidney injury and rapidly progressive glomerulonephritis [29,30]. In this study, neither lymphopenia nor low NLR were associated with classical complement activation.

There were several limitations concerning this study. One limitation was the cross-sectional design that included only a snapshot of measured values, without the ability to assess the kinetics of C1q-CIC and complement activation, especially in relation to alterations in kidney function. Furthermore, the lack of associated kidney biopsy samples did not allow us to correlate C1q-CIC levels with histological findings that could establish a connection with actual active AMR, although this issue remains controversial for graft function prediction after kidney transplantation [31]. Although we found that C1q-CIC levels were higher in patients with DSAs, we did not specifically detect C1q-binding DSAs, as this would require a different technique based on single antibody beads [3]. Thus, we were not able to, beyond doubt, attribute the higher C1q-CIC levels observed in DSA-positive KTRs to DSA-binding versus other unspecified reasons. Nevertheless, with the exception of three patients (one DSA (+) and two DSA (−) KTRs) who had a history of systemic lupus erythematosus, our cohort did not include any subjects whose medical history or cause of ESKD would be expected to trigger complement activation through the classical pathway. Thus, we believe that there is minimal, if any, chance that the classical complement pathway activation in our DSA-positive patients could be attributed to antibodies other than the anti-HLA DSAs. Of note, the prevalence of C1q-binding DSAs showed a wide range, from less than 15% to more than 60%, depending on the cohort [3,15,17,32]. Nevertheless, we consider that the significant correlation of MFI to C1q-CIC levels establishes a connection between at least the top end of MFI levels to the ability of DSAs to fixate C1q.

In conclusion, in this study we have shown that DSA-positive kidney transplant recipients have a higher serum concentration of C1q-CIC and increased classical pathway complement activation. This feature appears to carry the potential to contribute to more efficient risk stratification of KTRs, as far as future renal outcome is concerned. However, larger scale randomized studies are required, so that this marker can be established in clinical practice.

## 4. Materials and Method 

### 4.1. Patients

In this cross-sectional study we reviewed all KTRs actively monitored at the kidney transplantation outpatient clinic, with recently (within the previous 3 months) available laboratory determination of anti-HLA antibodies (*n* = 153). In all patients, DSAs were identified through comparison of donor–recipient HLA mismatch to the antibody profile in each patient. For all detected DSAs, the reported strength was based on the mean fluorescent intensity (MFI) of one solid-phase single-antigen bead (SAB) and in cases where more than one bead corresponding to the donor type was present within the panel, we recorded the corresponding bead with the highest MFI level. Following detection of KTRs with DSAs, we selected another group of matched KTRs without evidence of DSAs, of similar age and time after transplant operation. This latter group consisted of twice as many subjects as the KTRs with DSAs. Finally, we also used a third group of subjects (normal controls) with no previous history of kidney disease.

### 4.2. Ethics Approval and Consent to Participate

The study was approved by the hospital’s Ethics Committee and was in accordance with the Helsinki declaration as revised in 2013 (Approval number: 230/23.05.2024) and the declaration of Istanbul. A written informed consent was given by all participants, both KTRs and normal controls, before enrollment.

### 4.3. Immunosuppressive Treatment 

All KTRs followed the same immunosuppressive regimen. This included an IL-2 receptor blocker (basiliximab) as part of the induction therapy, methylprednisolone (500 mg intravenously on day zero followed by gradually reduced doses to 16 mg per os on day 30), a calcineurin inhibitor (cyclosporine or tacrolimus depending on their immunological risk), and mycophenolate mofetil (1.5–2 g/day).

### 4.4. Detection of Donor-Specific HLA Antibodies

LABScreen SAB assay (One Lambda, Canoga Park, CA, USA) on a Luminex platform was used to identify HLA specificities as per protocol. In this method, multiplexed microbeads, each coated with a single antigen, were incubated with patient serum and washed to remove unbound antibody. Then, anti-human immunoglobulin antibody conjugated to phycoerythrin was added and washed for a final time. Finally, the microbeads were examined in a flow analyzer (Luminex, Austin, TX, USA) and data were analyzed using HLA Visual software version 1.0. The cutoff for a positive reaction in IgG SAB assay was set at a normalized mean fluorescence intensity (MFI) value of 500 or greater. This value was chosen based on the proposed cut off value by Wisse BW et al. as well as the established DSA reporting standards of our laboratory [33].

### 4.5. Serum Creatinine Determination and eGFR Calculation

Serum creatinine values were measured with an automated analyzer, ADVIA^®^ 2400 Chemistry System (Siemens, Berlin, Germany). Estimated glomerular filtration rate (eGFR) was calculated according to the CKD-EPI formula, as indicated by the Kidney Disease: Improving Global Outcomes (KDIGO) guidelines [34].

### 4.6. Enzyme Immunoassays

Circulating C1q immune complexes were determined using the enzyme immunoassay (EIA) kit MicroVue CIC-C1q (Quidel, OH, USA). The kit is based on the principle that complement-fixing immune complexes will bind to immobilized human C1q purified protein. There are no established normal values for circulating C1q immune complexes. However, as mentioned in the manufacturer’s specifications sheet, mean ± SD values from normal control subjects are 2.1 ± 1.9 μg Eq/mL. The total classical complement pathway activity in human serum was quantified with the EIA kit MicroVue CH50 (Quidel, Athens, OH, USA), which is based on the complement activation followed by the measurement of terminal complement complexes (TCCs). The normal values for CH50 that are mentioned in the manufacturer’s specifications sheet are >70 CH50 U Eq/mL with mean ± SD values of 133 ± 54 CH50 U Eq/mL. Measurements were performed according to the manufacturer’s protocol. Serum samples were obtained after separation from clotted blood by centrifugation for 10 min at 1200× *g* in 4 °C. Samples were aliquoted and appropriately stored at −80 °C until assay.

### 4.7. Statistical Analysis

Results are expressed as mean ± SD. Normal or skewed distribution of continuous variables were analyzed using the Kolmogorov–Smirnov test. Differences of means of serum CIC-C1q, CH50, and eGFR levels between different groups of patients and controls were analyzed with the Kruskal–Wallis test with Dunn’s post-hoc analysis. Differences in means of serum CIC-C1q and CH50 among KTRs with preformed or de novo DSAs were analyzed with the Mann–Whitney test. Correlation between biochemical data and CIC-C1q levels was examined with Spearman’s test.

A two-sided *p* < 0.05 was considered as statistically significant. Statistical calculations were performed using SPSS, version 23.0 (SPSS Inc., Chicago, IL, USA) and GraphPad Prism (version 8.0.2 for Windows, GraphPad Software, San Diego, CA, USA).

## Figures and Tables

**Table 1:** Clinical, biochemical, and hematological features of patient and control groups.

	Healthy Subjects	Kidney Transplant Recipients	
		DSA-Negative	DSA-Positive
Number of subjects per group	22	28	15
Gender (M/F)	9/13	13/15	10/5
Age (years)	46 ± 14	55 ± 15	50 ± 11
Serum creatinine (mg/dL)	0.8 ± 0.1	1.4 ± 0.6	1.7 ± 0.8
eGFR (mL/min/1.73 m^2^)	106 ± 13	61 ± 27	53 ± 24
Neutrophil count (×10^3^/μL)	3.5 ± 0.8	4.8 ± 1.5	5.3 ± 2.6
Lymphocyte count (×10^3^/μL)	2.3 ± 0.6	2.2 ± 0.9	2.0 ± 0.7
Neutrophil/lymphocyte ratio	1.6 ± 0.6	2.6 ± 1.4	3.0 ± 1.7
Mean fluorescence intensity DSA (median, 25% and 75% percentile)	-	-	2443 (1683–6039)
Serum IgG levels (mg/dL)	-	966 ± 305	1087 ± 363
**Cause of ESKD**			
Glomerulonephritis	-	7	6
Diabetes mellitus	-	3	-
Hypertension	-	1	-
Alport syndrome	-	-	2
Tubulointerstitial disease		8	1
Unknown	-	9	6
**Immunosuppressant treatment**			
Glucocorticosteroids	-	28	15
Tacrolimus	-	25	15
Cyclosporine	-	3	
Mycophenolate	-	25	15
Everolimus	-	3	-

## Data Availability

Data are contained within the article and Supplementary Materials.

## Supplementary Materials

The following supporting information can be downloaded at: https://www.mdpi.com/article/10.3390/ijms252010904/s1.

## Abbreviations

DSA	Donor-specific antibodies
KTR	Kidney transplant recipient
AMR	Antibody-mediated rejection
CIC	Circulating immune complexes
CH50	Complement hemolysis 50%
MFI	Mean fluorescent intensity
NLR	Neutrophil-to-lymphocyte ratio
