# Supplementary material for: Circulating Immune Complexes and Complement Activation in Sensitized Kidney Transplant Recipients
Source: Int J Mol Sci. 2024 Oct 10;25(20):10904. doi: 10.3390/ijms252010904 (PMC11507516; doi:10.3390/ijms252010904)
Supplement: Supplementary file 1 [file ijms-25-10904-s001.zip › ijms-3218508-supplementary.pdf]

Supplemental Table S1. Comorbidities in kidney transplant recipients.

	DSA negative	DSA positive	p-value
Cardiovascular disease	7/28 (25%)	5/15 (33.3%)	0.723
Diabetes mellitus	10/28 (35.7%)	4/15 (26.7%)	0.735

Rejection episodes

Cases of biopsy proven rejection among the study participants are presented in supplemental table S2. No further cases of deterioration of renal function attributed to rejection ensued.

Supplemental Table S2. Kidney allograft biopsies reports and rejection episodes.

	DSA negative (N=28)	DSA positive (N=15)
Allograft biopsy	4	5
Antibody mediated rejection	0	4
T cell mediated rejection	2	1

All DSA (+) KTRs with biopsy proven AMR exhibited clinical and laboratory signs of rejection in the late post-transplant period (>12 months). The case of T-cell mediated rejection occurred in the first days after surgery. Both DSA (-) KTRs with biopsy proven T-cell mediated rejection experienced rejection in the early post-transplant period. Due to the very low number of biopsy proven rejection episodes, statistical analysis was not perforemed.
